# Membrane Cholesterol Modulates LOX-1 Shedding in Endothelial Cells

**DOI:** 10.1371/journal.pone.0141270

**Published:** 2015-10-23

**Authors:** Magda Gioia, Giulia Vindigni, Barbara Testa, Sofia Raniolo, Giovanni Francesco Fasciglione, Massimiliano Coletta, Silvia Biocca

**Affiliations:** 1 Department of Clinical Sciences and Translational Medicine, University of Rome “Tor Vergata, Via Montpellier 1, 00133, Rome, Italy; 2 Department of Systems Medicine, University of Rome “Tor Vergata”, Via Montpellier 1, 00133, Rome, Italy; University of Patras, GREECE

## Abstract

The lectin-like oxidized low-density lipoprotein receptor-1 (LOX-1) is a scavenger receptor responsible for ox-LDL recognition, binding and internalization, which is up-regulated during atherogenesis. Its activation triggers endothelium dysfunction and induces inflammation. A soluble form of LOX-1 has been identified in the human blood and its presence considered a biomarker of cardiovascular diseases. We recently showed that cholesterol-lowering drugs inhibit ox-LDL binding and internalization, rescuing the ox-LDL induced apoptotic phenotype in primary endothelial cells. Here we have investigated the molecular bases of human LOX-1 shedding by metalloproteinases and the role of cell membrane cholesterol on the regulation of this event by modulating its level with MβCD and statins. We report that membrane cholesterol affects the release of different forms of LOX-1 in cells transiently and stably expressing human LOX-1 and in a human endothelial cell line (EA.hy926). In particular, our data show that i) cholesterol depletion triggers the release of LOX-1 in exosomes as a full-length transmembrane isoform and as a truncated ectodomain soluble fragment (sLOX-1); ii) endothelial cells secrete a soluble metalloproteinase which induces LOX-1 ectodomain shedding and iii) long term statins treatment enhances sLOX-1 proteolytic shedding.

## Introduction

High levels of LDL cholesterol lead to atherosclerosis, increasing the risk of heart attack and ischemic stroke. Thus, LDL hypercholesterolemia is associated with the accumulation of oxidized LDL (ox-LDL), which is produced by oxidative stress and inflammation, and the growth of an unstable atherosclerotic plaque. The lectin-like oxidized low-density lipoprotein receptor-1 (LOX-1) is the major receptor of ox-LDL, it is expressed in various cells (including endothelial cells, macrophages and chondrocytes) and its expression is enhanced by proinflammatory cytokines [1,2]. LOX-1 plays a crucial role in endothelial dysfunction, characterized by reduced vasodilatation, proapoptotic and proinflammatory states and prothrombotic properties [3].

LOX-1 transmembrane receptor is a member of the C-type lectin-like protein family, it shows a type II orientation and its spatial organization is a critical step in signal transduction and receptor trafficking in cells. In human LOX-1, the lectin-like extracellular C-terminal domain (CTLD), which interacts with ox-LDL, forms a heart-shaped homodimer with an inter-chain disulfide bond at Cys140, not present in other species [4,5]. Mutations in the so-called basic spine region (located on top of the CTLD dimer) reduce LOX-1 binding affinity [4,5]. The NECK domain, connecting the transmembrane portion of the receptor to CTLD, is assumed to be a dimer consisting of two α-helices wrapped in a parallel coiled-coil structure [4,5,6]. Ligand-induced receptor clustering and association have a function in tuning the receptor activity. Thus, the dimeric form of the receptor has an intrinsic low affinity for ox-LDL, but multimerization and cluster organization in plasma membrane have been proposed to play a crucial role for the enhancement of LOX-1 activity [7,8,9,10] and a flexible NECK domain structure appears to facilitate the process of ox-LDL recognition [6].

Previous studies have shown that activation of LOX-1 by ox-LDL leads to a) up-regulation of LOX-1 expression at the cell surface and b) proteolytic ectodomain shedding from the cell surface with release of a soluble LOX-1 isoform (sLOX-1) [11,12]. A cleavage site in the NECK domain between Arg88 and Gln89 has been experimentally detected [13]. Although the significance of the soluble form of the receptor in circulating blood and the mechanisms of sLOX-1 release remain unclear, sLOX-1 appears to be a biomarker for acute coronary syndrome [14,15], for rheumatoid arthritis [16], for rupture of thin-cap fibroatheroma [17], for preeclampsia [18] and an early predictor of the metabolic syndrome [19]. Therefore, inhibition of LOX-1 activity indeed results in a protection against ox-LDL-mediated apoptosis and therapies directed against the activity of LOX-1 receptors may be effective in reducing the rate of atherosclerotic and inflammatory processes. In this respect, cholesterol-lowering drugs, such as 3-hydroxy-5-methylglutaryl coenzyme A (HMG-CoA) reductase inhibitors (collectively called statins) and methyl β cyclodextrin (MβCD), inhibit LOX-1 function by disrupting lipid rafts [20]. Of note, statins have been shown to reduce adverse clinical effects in patients with documented vascular events or at risk of them, even though several experimental and clinical evidences suggest that statins exert their beneficial effects also by other mechanisms beside the trivial lipid-lowering effect [21,22]. Thus, statins appear also to be able to inhibit LOX-1-mediated entry of ox-LDL inside human endothelial cells and rescue the ox-LDL-induced apoptosis [20]. More recently, we have demonstrated that statins, besides their indirect activity on LOX-1 activity, inhibit LOX-1 by direct interaction with the CTLD recognition domain [23].

Further, an important process (connected to the cholesterol-dependent regulation of LOX-1 activity) is its shedding by proteolytic enzymes with the production of sLOX-1. Several proteases mediate the pericellular proteolysis, leading to release of ectodomains in the extracellular space, including secreted matrix metalloproteinases (MMPs), membrane-anchored MMP (MT-MMPs), a disintegrin and metalloprotease domain (ADAM), a disintegrin and metalloprotease domain with throbospondin motifs (ADAMTS), cysteine and serine proteases [24,25,26,27,28]. Several metalloproteinase-dependent shedding processes are modulated by cholesterol levels [29,30,31,32], which indeed affect cell membrane organization. Thus, cell membrane cholesterol content was found to be critical not only in tuning receptor clusterization [20,33], but also in regulating the shedding of some membrane proteins, such as alpha secretase [29,34], human interleukin-6-receptor [35] and CD30 [36]. In this respect, it is important to point out that, also in fibroblasts, cell-membrane cholesterol depletion induces the activation of gelatinase proMMP-2 [37]. Previous reports suggest that LOX-1 also induces vascular extracellular matrix degradation through matrix metalloproteinases (MMPs) [38,39,40] and MMPs play a pivotal role in cardiovascular inflammation processes, with MMP-1, MMP-2 and MMP-3 being those most frequently correlated with a cardiovascular risk in humans [39,40,41]. Further, immunocytochemistry studies have demonstrated that MMP-1, MMP-9 and MMP-3 are expressed in atheromatous endothelial cells but not in cells present in normal arteries, pointing also out a significantly enhanced collagenolytic activity in atherosclerotic plaques [42]. It has been found that MMP-9 co-localizes with LOX-1 in vulnerable plaque [43]. MMPs proteolytic activity can be regulated at different levels and, endogenously, by tissue inhibitors of metalloproteinase (TIMPs) [25,40,44], which play an important role in keeping MMPs in a latent form and preventing their excessive activation and/or activity/ies. Indeed, many experimental studies have reported an unbalanced expression and activity of MMPs and TIMPs in a variety of cardiovascular diseases [40,45,46]. Together with changes of cardiac function, it has been shown that TIMP-1 interacts with MMP-2 and MMP-9 in patients with heart failure [47]. In particular, an increase of TIMP-1 appears associated with the raising of circulating levels of MMP-2 and MMP-9, not only in patients at the final stage of heart failure (or in the acute state of decompensation), but also in patients with subclinical heart failure [48].

Here we investigated the effect of cell membrane cholesterol level on the release of LOX-1 and analyzed the metalloprotease-dependent shedding of LOX-1 ectodomain in transfected cells (COS and HEK cell lines) and in a human permanent vein umbilical endothelial cell line (EA.hy926). In particular, in HEK-293 cell line (clone #19), stably expressing LOX-1 receptors, we have detected, for the first time, that LOX-1 is released as a full-length form in exosomes. On the other hand, in COS and endothelial cell lines, the main event is the release of the truncated isoform sLOX-1. Our findings indicate that a decrease in plasma membrane cholesterol by MβCD or statin treatment leads not only to down-regulation of LOX-1 in lipid rafts [20] but also to an enhanced shedding of LOX-1 ectodomain. The sheddase activity of ligand-bound and/or free LOX-1 receptors is carried out *in vitro* by a secreted MMP (*e*.*g*., MMP-1 and/or MMP-2).

## Materials and Methods

### DNA construct

For the expression in mammalian cells, human LOX-1 was subcloned into pEF/V5-His vectors (Invitrogen, Paisley, UK) as previously described [7].

### Cell cultures and transfection

COS cells, HEK-293#19 [20] and CHO-F2 clones stably expressing LOX-1-V5, and the human EA.hy926 endothelial cell line [49] were grown in DMEM (Dulbecco’s modified Eagle’s medium) (Biowest, Miami, FL) supplemented with 10% fetal bovine serum (Gibco, Paisley, UK), L-glutamine 1mM (Sigma Aldrich, St. Louis, MO), sodium pyruvate 1mM (Biowest, Miami, FL) and 100U/ml penicillin-streptomycin (Euroclone, Devon, UK). COS cells were transiently transfected with JetPEI (Polyplus Transfection, Illkirch, FR), following the manufacturer’s instructions, with a DNA/transfectant reagent ratio (w/v) of 1:2.

### Chemicals

Filipin, MβCD, glimepiride and atorvastatin were purchased from Sigma-Aldrich (St. Louis, MO). Lovastatin (Enzo Life Sciences Inc., Farmingdale, NY) was activated by the addition of 0,1 N NaOH (lovastatin/NaOH 2:3 v/v), at 50°C and neutralized with 1 N HCl to pH 7,2.

Phenylmethylsulfonylfuoride (PMSF) (Euroclone, Devon, UK), pepstatin A (Boehringer, Mannheim, DE), GM6001 inhibitor (Biomol Research Lab Inc.), protease inhibitor cocktail set III (AEBSF 100mM; aprotinin 80μM; bestatin 5mM; E-64 1,5mM; leupeptin 2mM; pepstatin A 1mM) (Calbiochem, La Jolla, CA) were used for specific protease class inhibition experiments.

Ox-LDL preparation, labelling and fluorescence analysis

Human LDL was prepared from fresh healthy normolipidemic plasma of volunteers by ultracentrifugation [50] and *in vitro* oxidized as described (Matarazzo et al., 2012). Ox-LDL was labelled with 1,1-dioctadecyl-3,3,3,3-tetramethyllindocarbocyanine perchlorate (DiI, Invitrogen, Paisley, UK) [7,51]. DiI-ox-LDL binding assay was performed as previously described [13].

### Medium fractionation

Exosome-associated proteins released in cell culture supernatants were isolated by sequential centrifugation steps, as described [52,53]. After clearing from cell debris, 250μl of conditioned media derived from confluent cells at a concentration of 0.8-1x10^6^ cells/ml were pelleted at 100,000 g to obtain pellet (P100) and supernatant (S100) fractions. S100 fractions were precipitated with 10% trichloroacetic acid (Sigma Aldrich, St. Louis, MO).

### Western blot analysis

Cell extracts and conditioned media of transfected COS cells were prepared as described [20], TCA precipitated and analyzed by SDS-PAGE gel electrophoresis in 10% or 12% polyacrylamide gels and transferred to polyvinylidene difuoride (PVDF) or nitrocellulose membranes (GE Healthcare, Chalfont St. Giles, UK). MAb anti-V5 IgG (Invitrogen, Paisley, UK), polyclonal anti-LOX-1 antibody (Aviva System Biology), anti-flotilin (Santa Cruz Biotechnology Inc., Santa Cruz, CA), anti-MMP-1 MaxPab mouse polyclonal antibody (Abnova, Taipei, Taiwan), anti-TIMP-1 mouse polyclonal antibody (Santa Cruz Biotechnology, Inc.), were used as primary antibodies. Goat anti-mouse IgG HRP and donkey anti-rabbit IgG HRP (Jackson Immunoresearch Laboratories Inc.; West Grove, PA; AbCam, Cambridge, UK) were used as secondary antibodies. Immunoreactive bands were detected with ECL LiteAblot Turbo (Euroclone, Devon, UK) and Novex ECL HRP Chemiluminescent substrate reagent kit (Invitrogen, Paisley, UK) at one-to-one ratio according to manufacturer’s instructions.

### Zymography

Confluent cells layers were washed three times with room temperature (rt) PBS buffer. Media were replaced by serum-free DMEM and cells were incubated at 37°C overnight (with or without ox-LDL), before cholesterol depletion treatments. Cells conditioned media were 10-times concentrated by speed-vac centri vapor. Protein concentrations were determined by Bradford assay and confirmed by running SDS-PAGE gel electrophoresis in 10% polyacrylamide gels prior to zymography. Concentrated medium samples were diluted in SDS-polyacrylamide gel electrophoresis sample buffer under non reducing conditions without heating. Samples were separated by 10%-SDS-polyacrylamide gels co-polymerized with 1mg/ml gelatin type B (Sigma Chemical Co.). After electrophoresis, gels were washed twice for 30 minutes in 2.5% Triton X-100 at rt and incubated overnight in “activity buffer” (50mmol/L Tris-HCl, pH 7.5, 10mmol/L CaCl2, 150mmol/L NaCl) at 37°C. As a control, incubation of gelatin gel with the metalloproteinase inhibitor (1mM GM6001) completely abolishes the appearance of the clear transparent band. Gels were stained in Coomassie Blue R 250 (Bio-Rad, Milan, Italy) in a mixture of methanol:acetic acid:water (4:1:5) for 1 hour and destained in the same solution without dye. Gelatinase activities were visualized as distinct bands, indicating proteolysis of the substrate. Recombinant human MMP-2 and MMP-9 (R & D System, Boston, USA) were used as a reference standard for proMMP-2 and proMMP-9.

### MTS assay

Cell cytotoxicity was evaluated by using the CellTiter 96 AQueous One Solution Reagent (Promega) containing a tetrazolium compound [3-(4,5-dimethyl-2-yl)-5-(3-carboxymethoxyphenyl)-2-(4-sulfophenyl)-2H-tetrazolium, inner salt; MTS] and an electron coupling reagent (phenazine ethosulfate; PES). Confluent EA.hy926 cells plated in 96 well tissue culture plates (BD Falcon) were washed three times in PBS and incubated in serum free medium with different concentration of ox-LDL. MTS assay was performed by adding 10μl/well of reagent and absorbance at 490nm was recorded using an ELISA plate reader.

### Crossed-cells assay for LOX-1 shedding induced by secretome

In the crossed-cells assay, the cell lines employed were: i) human endothelial EA cells (as a source of secretome), and ii) COS cells transfected with a V5-tagged-LOX-1 (as general “living” plasma membranes harbouring receptor LOX-1). COS cells were transfected with the full-length human LOX-1 cDNA (containing an engineered V5 tag), and LOX-1 expression assessed by Western blot. 70–80% confluent EA cells were washed 3 times in PBS, and incubated overnight with serum-free DMEM, followed by harvesting of EA secretome. Conditioned media were cleared from cellular debris by centrifugation at 1,500 rpm for 10 minutes, centrifuged at 10,000 g for 30 min and immediately added to LOX-1-V5 transfected COS cells for 10 min at 37°C. The secretome exposed to COS cells was harvested, TCA precipitated, suspended in a volume 10 times more concentrated and probed by Western blot with anti-V5 antibodies. This experimental design reveals only sLOX-1 coming from COS cells (which express the anti-V5 tagged LOX-1) and not from EA cells (which express endogenous LOX-1).

### Sheddase assay of LOX-1 receptor

COS cells were transfected with LOX-1-V5 cDNA at 37°C overnight (as reported above). To remove all sets of secreted proteases, cells-layers were washed 3 times with PBS buffer, and then living cells were incubated with or without 30μM soluble active MMP in phosphate buffer for 1 hour, at 37°C. The stoichiometry concentration of active enzyme was titrated *in vitro* by fluorimeter employing MMP-specific fluorogenic substrate (Mca-Pro-Leu-Gly-Leu-Dpa-Ala-Arg-NH_2_, Calbiochem) and a broad spectrum hydroxamate inhibitor (GM 6001) (BIOMOL International, Enzo Life Sciences), as previously described [54].

To detect the intact LOX-1 on cell surface, the enzymatic treatment was followed by incubation with a saturating concentration of fluorescent labeled Dil-ox-LDL, for 1 hour at 4° C, as previously described [20]. The effect of LOX-1 proteolysis at the cell surface was evaluated by looking at the disappearance of intact LOX-1 receptors on single cell by cyto-fluorescence technique. The quantification was performed by counting Dil-oxLDL-stained cells over the numbers of blue (Hoechst 33342) stained nuclei (n>150 cells). As a positive control, we have used the metalloproteinase-dependent shedding of LOX-1 induced by MβCD.

### Image processing and densitometric analysis

Images of zymography gels and western blot films were digitized by scanning. Bands were analyzed by using Molecular Analyst software (Bio-Rad Laboratories) and ImageJ software, respectively. Background measurements were subtracted from the integration area measured for each band. Thus, results were standardized for each gel and expressed in dimensionless units.

### Statistical analysis

Data were analyzed using one-way analysis of variance. Intergroup difference were determined by the Student-Newman-Keuls test. Results are expressed as a mean ± S.E.M. Differences were considered statistically significant when P < 0.05 (*), P < 0.01 (**) or P < 0.001 (***).

## Results

### Membrane cholesterol regulates LOX-1 release in transfected cells

**Fig 1:**
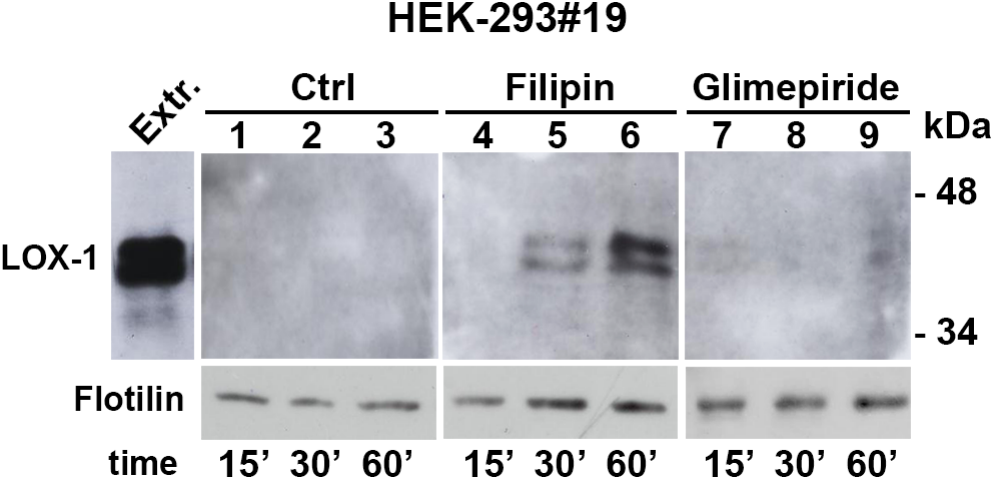
LOX-1 release in exosomes. HEK-293#19 cells stably expressing LOX-1 were treated with filipin (5μM) and glimepiride (5μM) for 15, 30 and 60 min. Total protein extract (5μg) was loaded as a positive internal control of electrophoretic mobility (Extr.). Anti-V5 antibody was used to detect LOX-1-V5 protein and antibody directed against flotilin to verify equal protein loading.

We have studied LOX-1 expression and release in different cell types, such as HEK-293, CHO and COS cells. Notably, differences in the level of soluble LOX-1 (sLOX-1) have been detected among different cell lines. In particular, HEK-293 has a reduced extent of sLOX-1 production. To verify whether LOX-1 is secreted also as full-length form in exosomes, we have analyzed the conditioned medium derived from the cell line HEK-293 (clone #19), stably expressing human LOX-1 receptors, grown in the presence of lipid raft-interfering drugs, such as glimepiride and filipin. Glimepiride activates GPI-phospho-lipase C, which induces redistribution of lipid-raft resident proteins. Filipin, instead, is a polyene antibiotic that binds to lipid raft and non-raft membrane cholesterol [55] and co-localizes with LOX-1 receptors *in vivo* [20]. In order to study full-length LOX-1 release in exosomes, cells were grown for 15, 30 and 60 min in DMEM in the presence of glimepiride and filipin (both drugs at 5μM concentration) and, after clearing from cell debris, conditioned media were pelleted at 100,000 g to isolate exosomes. For each treatment pellets (P100), corresponding to 250μl of conditioned medium (from confluent cells at a concentration of 0.8-1x10^6^ cells/ml), were analyzed by Western blotting. Fig 1 shows that full-length LOX-1 receptors, derived from extracts of HEK-293#19, run at 46kDa (Fig 1, extract). Exosome-bound LOX-1 was not detectable in samples derived from non-treated cells (Fig 1, lanes 1–3) and only barely detectable in cells incubated with glimepiride (Fig 1, lanes 7–9). Conversely, treatment with filipin induces release of exosome-associated LOX-1 in a time-dependent manner (Fig 1, lanes 4–6). Of note, LOX-1 runs as a doublet band in some experimental replicates, due to a different extent of glycosylation [7]. Antibody directed against flotilin, an exosomal marker protein, was used as normalization factor. The analysis of S100 supernatant fractions derived from the same amount of conditioned medium (250μl) has shown that the soluble truncated LOX-1 (sLOX-1) is not detectable, even when HEK-293#19 clone was treated with filipin (not shown).

**Fig 2:**
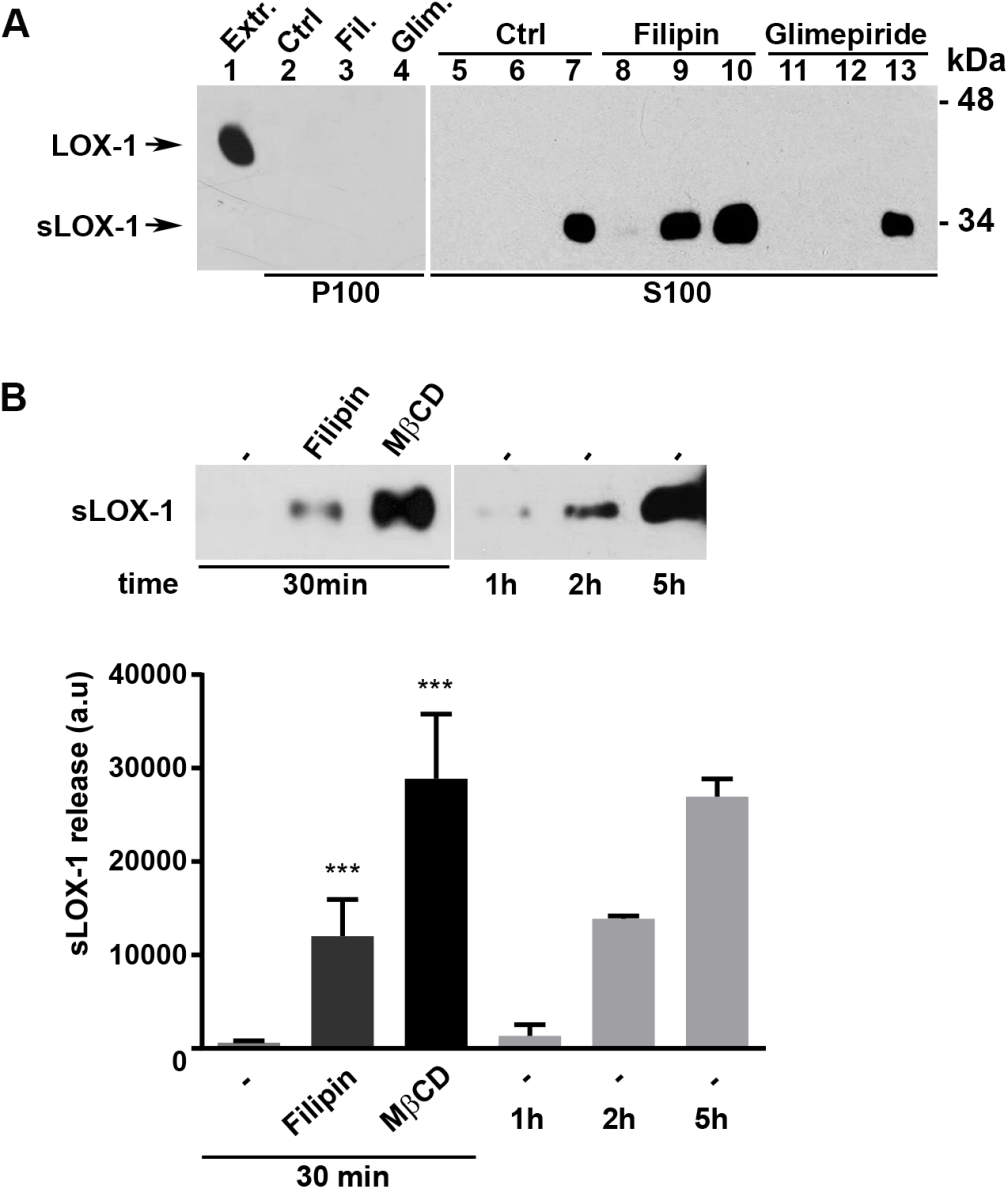
sLOX-1 release in COS cells. (A) LOX-1-V5-COS transfected cells were treated with filipin (5μM) and glimepiride (5μM) for 15, 30 and 60 min. Conditioned media were centrifuged at 100,000 g and the resulting pellets (P100, lanes 2–4) and supernatants (S100, lanes 5–13) were analyzed by Western blotting. Lane 1 (Extr.) shows total protein extract (5 μg) loaded as a positive internal control of electrophoretic mobility of full-length LOX-1 receptor. (B) Upper panel shows sLOX-1 amount released in cells treated with Filipin (5μM) and MβCD (5mM) for 30 min compared to sLOX-1 constitutively released from untreated cells at 1, 2 and 5 hours. Lower panel shows the densitometric analysis for sLOX-1 band intensity. Data in histograms represent the average ± SEM of three experiments, P < 0.05 (*), P < 0.01 (**) or P < 0.001 (***).

We further studied LOX-1 removal from cell membranes in transiently transfected COS cells, where LOX-1 expression is 10-fold higher than in LOX-1 expressing HEK-293 stable clones. The released LOX-1 in the two fractions (*i*.*e*., P100 pellet and S100 supernatant), derived from the same amount (250μl) of conditioned medium was analyzed (Fig 2A). In this cell model system, only a very small percentage of LOX-1 (≤ 5%), barely detectable, is exosome-bound (Fig 2A, lanes 2–4) and most of LOX-1 is shed as a truncated form of MW of 32–34 kDa released in the medium (S100 fractions, lanes 5–13). It is interesting to remark that also in LOX-1 expressing COS cells filipin treatment, but not glimepiride, induces a higher extent of sLOX-1 release, as compared to the control, indicating that membrane cholesterol level exerts an important control on LOX-1 secretion.

In COS cells, we have further studied the role of membrane cholesterol on LOX-1 ectodomain shedding, by using 5mM Methyl-β-ciclodextrin (MβCD) for 30 min. This treatment decreases membrane cholesterol by approximately 40–50% [20], specifically extracting cholesterol from the plasma membranes and therefore disrupting lipid-rafts and caveolae [56]. As it is shown in Fig 2B (upper panel), MβCD treatment results in a massive release of sLOX-1 in the medium. By densitometric analysis, the amount of sLOX-1 released by MβCD treatment in 30 min is 3 times more than that resulting from filipin treatment and it is comparable to the amount of sLOX-1 released in conditioned medium of not treated cells in 5 hours (Fig 2B, lower panel). Of note, the phenomenon is reversible as increasing membrane cholesterol by cholesterol-loaded MβCD (cholesterol water soluble) (200 or 400μg/ml) leads to a marked decrease of the amount of sLOX-1 shedding and its release in the medium (data not shown).

### Long-term treatment with statins enhances LOX-1 shedding

**Fig 3:**
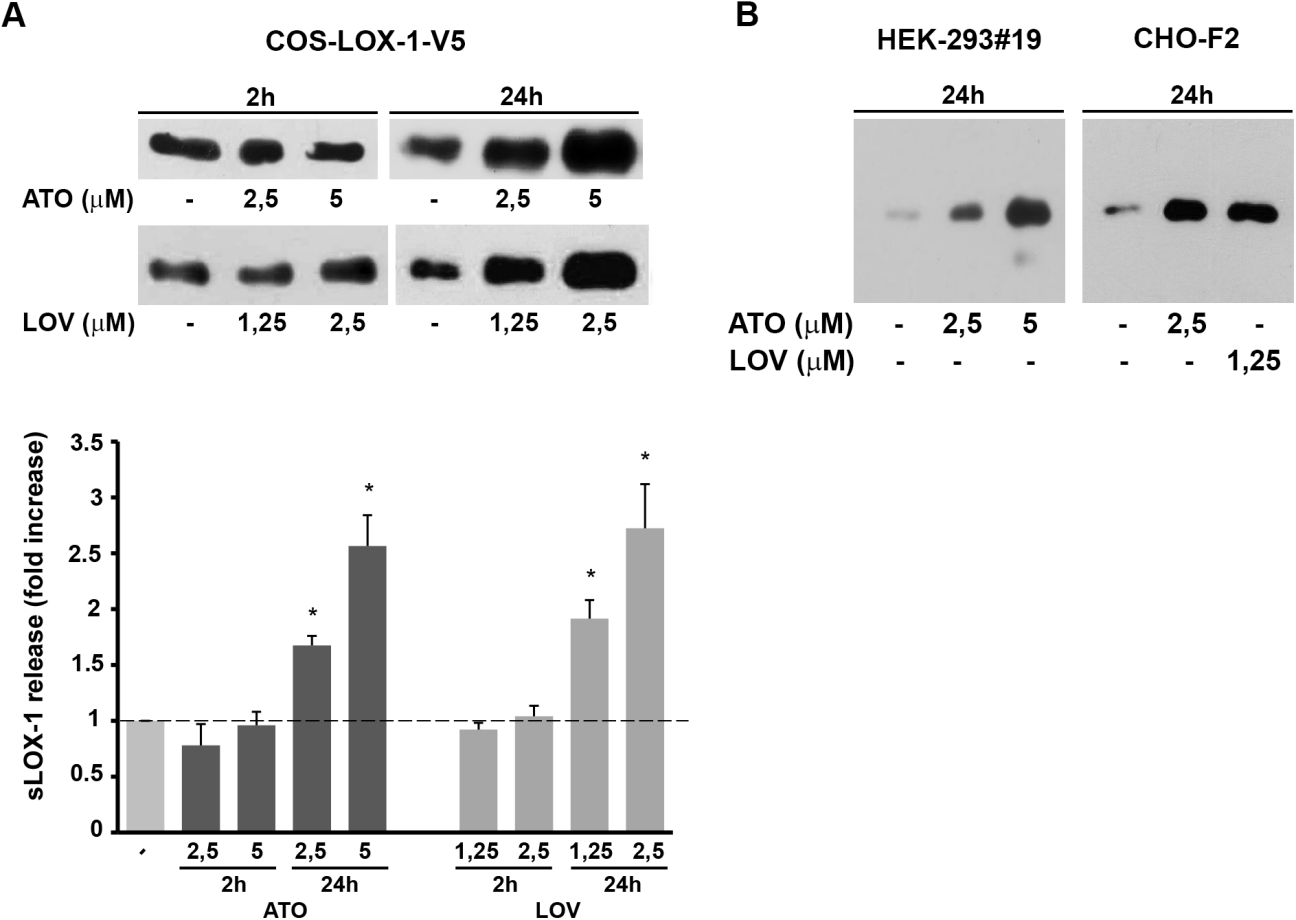
Effect of statins on LOX-1 shedding. (A) Upper panel shows short and long term treatments of LOX-1-V5 COS transfected cells with atorvastatin (2.5 and 5μM) and lovastatin (1.25 and 2.5μM). Densitometric analysis of fold change of protein band of sLOX-1 over respective time matched controls (lower panel). Data represent the averages ± SEM of three experiments. P < 0.05 (*). (B) Statins treatments of HEK-293 #19 and CHO-F2 cells incubated in serum free medium in the presence of atorvastatin (2.5 and 5μM) and lovastatin (1.25μM) for 24h at 37°C.

Statins inhibit cholesterol biosynthesis and are largely employed in clinics for their effects on lowering total and circulating low-density lipoprotein-cholesterol (LDL-C). To investigate whether statins behave “in vitro” similarly to MβCD, reported above, we compared sLOX-1 levels by analysing 250μl of conditioned media derived from the same number of cells treated or not with atorvastatin and lovastatin. Fig 3A shows that, after 24 hours statin treatment, LOX-1 ectodomain shedding is enhanced in LOX-1-V5 transiently transfected COS cells in a dose-dependent manner. Densitometric analysis indicates that either 5μM atorvastatin or 2.5μM lovastatin leads to 1.5÷1.7 fold increase in LOX-1 shedding (Fig 3A, lower panel), respectively. A similar result is observed in steadily LOX-1 expressing HEK-293#19 and CHO-F2 clones incubated with atorvastatin and lovastatin (Fig 3B). Therefore, there is a consistent pathway connecting exposure of cells to statins, reduction of membrane cholesterol content and enhancement of LOX-1 shedding by a proteolytic enzyme, with the extracellular release of sLOX-1. Of note, no significant changes in the amount of sLOX-1 released in conditioned media were observed after 2 hours treatment with statins (Fig 3A), indicating that the effect observed is not due to direct interaction between LOX-1 and statins [23].

### sLOX-1 shedding is inhibited by MMP inhibitor GM6001

**Fig 4:**
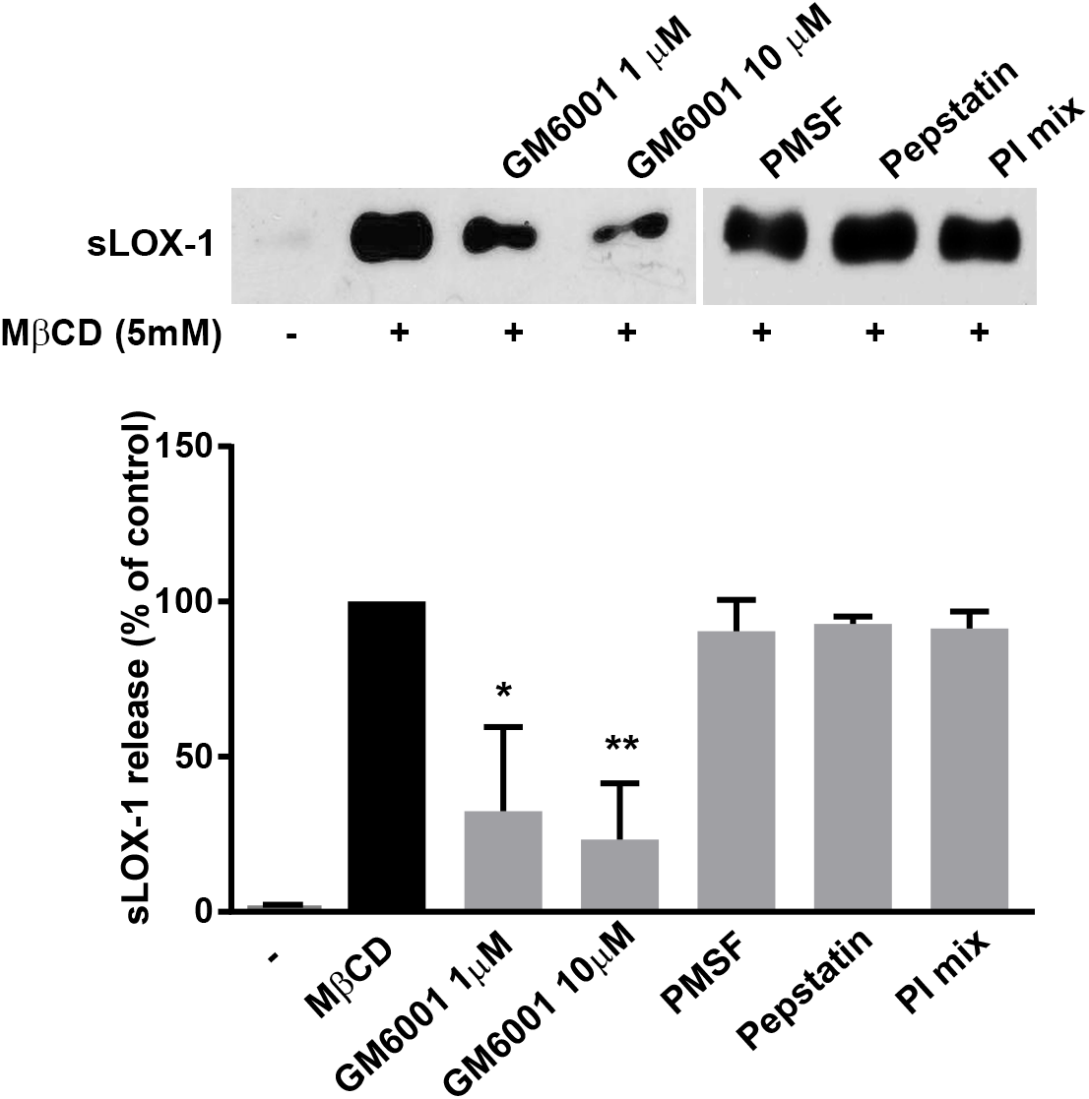
Western blot of sLOX-1 released from cells treated with MβCD and different protease inhibitors. LOX-1-V5-COS transfected cells were incubated with MβCD, GM6001, PMSF, Pepstatin A and the protease inhibitor cocktail (PI mix) (Calbiochem), as indicated (upper panel). Histogram shows the densitometric analysis expressed as percentage of reduction of sLOX-1 released in the presence of different protease inhibitors. 100% refers as sLOX-1 released in the presence of 5mM MβCD for 30 min (lower panel). Data in histograms represent the average ± SEM of three experiments, P < 0.05 (*), P < 0.01 (**) or P < 0.001 (***).

To identify the class of proteolytic enzymes responsible of LOX-1 ectodomain shedding we screened different inhibitors for their effects on sLOX-1 release from COS cells treated or not with MβCD for 30 min. As it can be seen in Fig 4 (upper panel), the broad-spectrum metalloproteinase inhibitor GM6001 impairs LOX-1 shedding in a dose-dependent manner. No statistically significant effect was observed with the serine (PMSF 2mM), aspartic (Pepstatin A 10μM) protease inhibitors, with the protease inhibitor cocktail (Calbiochem, La Jolla, CA) (1:1000 dilution), which contains 6 different inhibitors with broad specificity for aspartic, cysteine, serine proteases and aminopeptidases. These results demonstrate that LOX-1 ectodomain shedding is mediated by at least one of the member of the metalloproteinase class.

### Membrane cholesterol regulates LOX-1 shedding in endothelial cells

**Fig 5:**
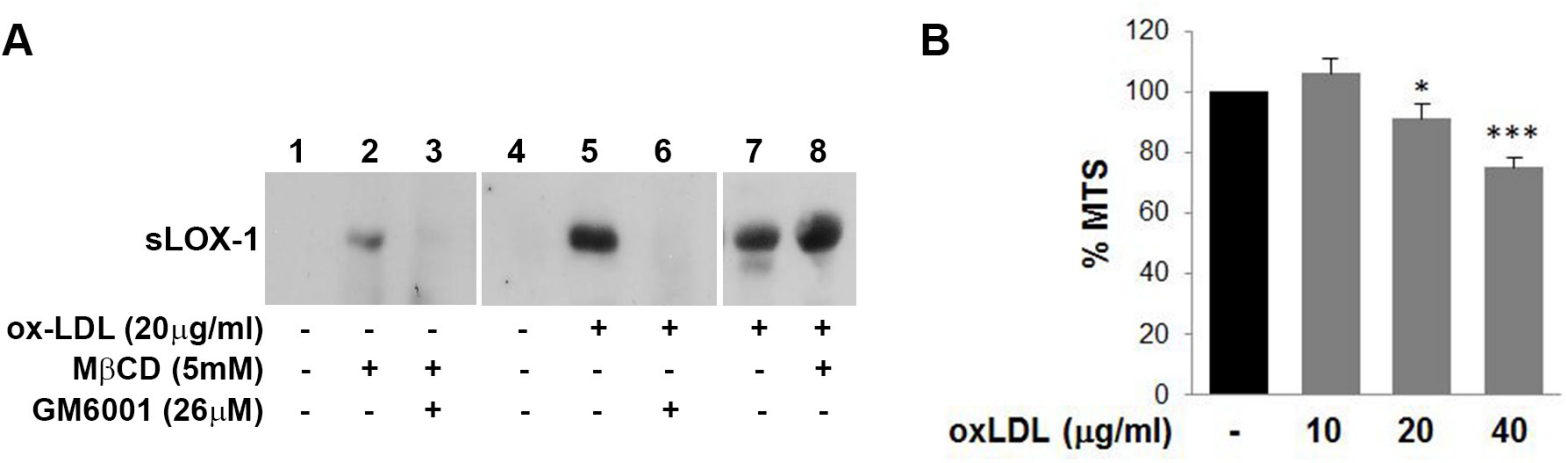
sLOX-1 release in EA.hy926 cells. (A) EA.hy926 cells were grown overnight in serum free medium in the absence (lanes 1–4) or in the presence (lanes 5–8) of ox-LDL (20μg/ml). MβCD was added to cells for the last 30 min incubation (lane 2, 3 and 8). GM6001 inhibitor was present for 30 min (lane 3) or overnight (lane 6). Western blot analysis of conditioned media was performed with anti-human LOX-1 receptor. (B) MTS assay performed on EA.hy926 cells plated at a density of 1,5x10^4^ cells/well and incubated overnight with different concentration of ox-LDL. Results are expressed as percentage of absorbance ± SEM compared to control cells.

To analyze the biological significance of sLOX-1 shedding and the effect of cholesterol modulation, we have employed the human umbilical vein endothelial cell line EA.hy926 (EA) as a model system [49], since ox-LDL-induced over-expression of LOX-1 receptors is associated to endothelial dysfunction [2]. LOX-1 protein constitutive expression is very low in EA cells, but it becomes significantly up-regulated upon exposure to ox-LDL. Like any endothelial cell, EA cells respond to ox-LDL by inducing cell shrinkage, a typical feature of cell death [57,58,59,60]. We have therefore analysed the cytotoxic effect of different concentrations of ox-LDL by MTS cell viability assay. Overnight exposure to different ox-LDL concentrations, ranging from 20 to 40μg/ml, decreases the MTS signal by 10±4% and 30±6%, respectively, as compared with untreated cells (Fig 5B). Fig 5A, lanes 5 and 7 shows that exposure of EA cells to 20μg/ml concentration of ox-LDL for 24 hours gives rise to an enhancement of LOX-1 expression without any apparent EA cell damage and this is accompanied by a marked release of sLOX-1 in the medium; therefore, this concentration was employed for screening inhibitors of sLOX-1 shedding. Although sLOX-1 is barely detectable in not treated cells (Fig 5, lanes 1), a clear band of sLOX-1 is visible when endothelial cells are treated with MβCD for 30 min (Fig 5, compare lanes 1 with lane 2). Similarly to COS cells, the metalloproteinase inhibitor GM6001 inhibits sLOX-1 shedding in EA cells, completely abolishing its release at 26μM concentration (Fig 5, lanes 3 and 6). Taken together, these results indicate that LOX-1 is abundantly shed in endothelial cells and, as observed in COS transfected cells, membrane cholesterol level regulates release of LOX-1. No visible trace of the whole receptor form associated to exosomes was detected in EA cells secretome.

### MMPs proteolytic activity of EA cells conditioned media

**Fig 6:**
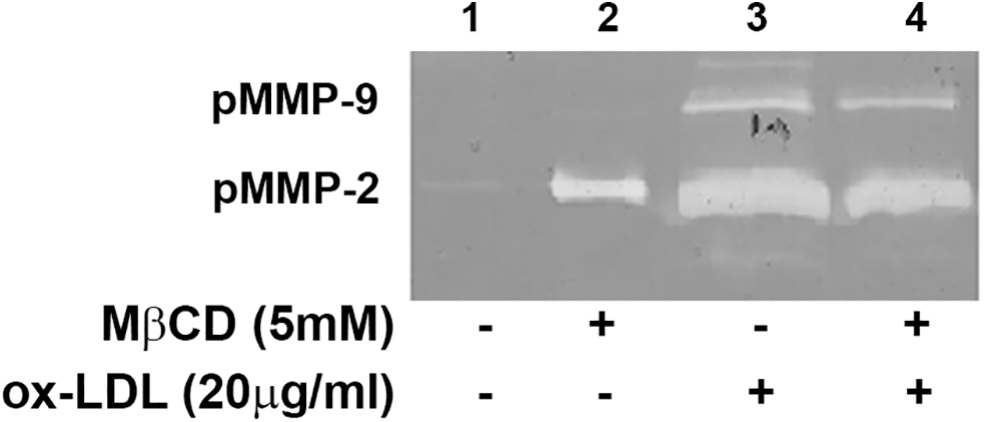
Zymographic analysis of EA cells conditioned media exposed to ox-LDL and/or MβCD. Bands corresponding to the proenzyme forms of gelatinases proMMP-2 (lanes 1–4) and proMMP-9 (lanes 3 and 4) were clearly detected in the conditioned media of treated cells. The gel shown is representing zymograms from 4 independent experiments.

The presence of matrix degrading proteases in EA cells-conditioned media was analyzed by gelatin zymography (Fig 6), where proteolytic transparent bands, corresponding to proMMP-9 and proMMP-2, were observed. The presence of lower molecular weight forms can be attributed to the activated forms of both enzymes. As a control experiment, incubation of gelatin gels with 1mM GM6001 completely abolished the appearance of gelatinolytic clear bands (not shown), indicating that each gelatinolytic band indeed belongs to this MMP class of proteases. In details, Fig 6 shows the presence of proMMP-2 under each experimental condition, its production being enhanced by cells treatment with ox-LDL and/or MβCD (lanes 2–4). Densitometric analysis of lytic bands (lane 1 versus lane 3) revealed that ox-LDL-treated secretome exerts 50 times higher gelatinolytic activity with respect to control secretome. Further, untreated EA cells did not secrete proMMP-9 (lane 1), whereas ox-LDL addition leads to secretion of proMMP-9 in the medium (lanes 3 and 4). Exposure of EA cells to ox-LDL induced the production and/or the activation of both MMP-2 and MMP-9, that might be involved in shedding of sLOX-1.

### Oxidized LDL differently regulates MMP-1 and TIMP-1 secretion in EA cells

**Fig 7:**
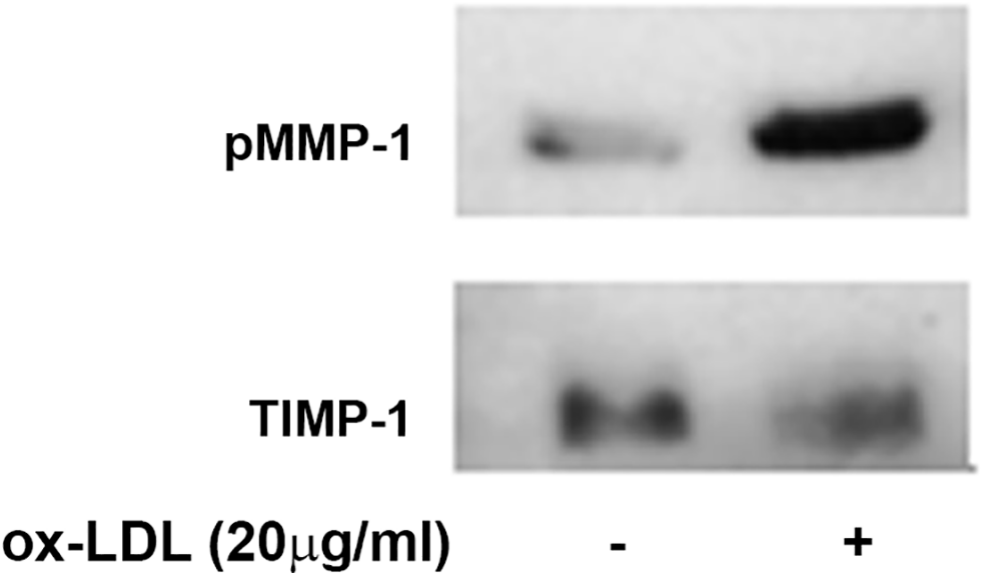
Immunoblotting of EA cells conditioned media probed with anti MMP-1 polyclonal mouse antibodies (upper panel), and TIMP-1 polyclonal mouse antibodies (lower panel). Optical density analysis was used to semi-quantify the protein secretion levels upon ox-LDL cell-treatment.

Secretion of MMP-1 and TIMP-1 proteins by EA cells exposed to 40μg/ml ox-LDL was determined by immunoblotting analysis of EA cells-conditioned medium by anti MMP-1 and TIMP-1 antibodies, respectively. Semi-quantitative analysis of bands from Fig 7 (upper panel) shows that ox-LDL incubation induces about 3-fold increase of MMP-1 secretion (2.7±0.4 fold increase). Conversely, the TIMP-1 levels are more abundant in the control than in the ox-LDL treated sample (1.5±0.025 folds)(see Fig 7, lower panel), showing that TIMP-1 secretion was significantly down-regulated upon exposure to ox-LDL. Overall, the enhancement of the MMP proteolytic activity of EA cell secretome appears to be related to an increase of MMPs/TIMPs ratio in response to ox-LDL stimuli.

### EA cells secretome prompts sLOX-1 shedding

**Fig 8:**
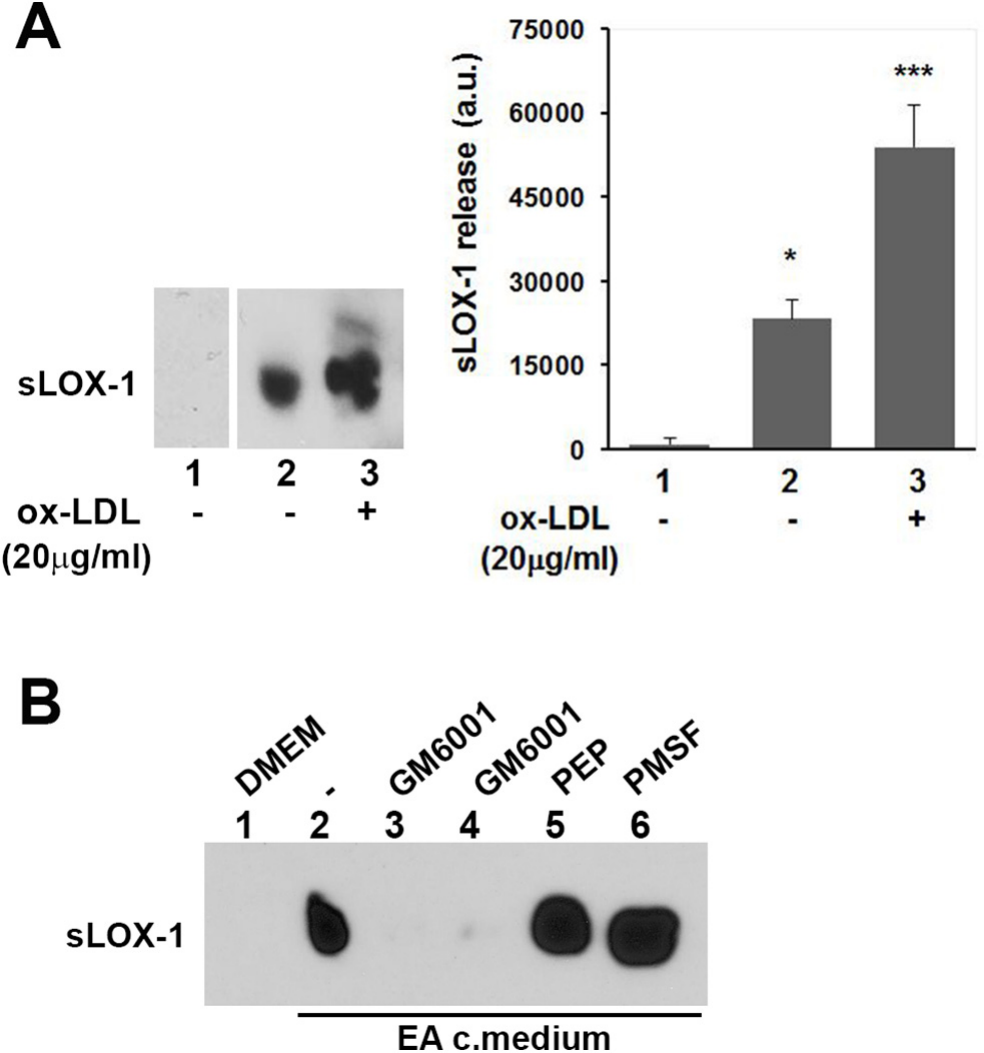
EA.hy926 cells secretome induce LOX-1 shedding. (A) Western blot analysis of conditioned medium derived from cross-cells experiments. LOX-1-V5 COS transfected cells were incubated for 10 min with DMEM (lane 1), medium derived from untreated EA cells (lane 2) and medium derived from EA cells conditioned in the presence of ox-LDL (20μg/ml) (lane 3). The histogram on the right shows the densitometric analysis of sLOX-1 band intensity. (B) Effect of different classes of protease inhibitors on LOX-1 shedding. Inhibitors were added during the 10 min final incubation time. GM6001 was used at 2.6 and 26μM (lanes 3 and 4), Pepstatin A at 10μM (lane 5) and PMSF at 2mM (lane 6).

Ox-LDL-mediated up-regulation of MMP-1, MMP-2 and MMP-9 and the concurrent down-regulation of TIMP-1 in EA cells secretome suggests that the proteolytic shedding of sLOX-1 can be triggered by a soluble secreted mediator. Hence, to validate whether the human endothelial EA cells release (a) soluble factor(s) which may bring about sLOX-1 shedding, we performed crossed-cells experiments employing EA cells as a source of biologically relevant secretome, and COS cells transfected with V5-tagged-LOX-1 (LOX-1-V5) to provide a functional membrane receptor LOX-1. Beside the potential soluble sheddase(s), EA cells secretome contains also sLOX-1 produced by the set of proteases of EA cells during the incubation time. Therefore, the experimental setup was designed so as to reveal by Western blot the sLOX-1 derived from COS cells (by anti-V5 antibody) but not from EA cells. EA cells conditioned media were prepared by incubating thoroughly washed cells with serum–free DMEM and harvesting the conditioned medium after 18 hours. Centrifuged media were incubated for 10 min with LOX-1-V5 transfected COS cells. Fig 8 reports the Western blot probed with anti-V5 antibodies and it shows that the shedding activity increases upon exposure of COS cells to the EA cells secretome. Thus, while COS cells incubated with fresh DMEM medium did not yield any detectable amount of sLOX-1 (lanes 1), conditioned medium from endothelial cells shed very high levels of sLOX-1 from COS cells (lanes 2), clearly indicating that EA endothelial cells secrete a factor which enhances sLOX-1 shedding. To investigate whether the sLOX-1 sheddase activity of EA secretome was strengthened by the proteases induced by ox-LDL, we collected conditioned media after treating EA cells for 18 hours with 20μg/ml ox-LDL. The amount of sLOX-1 released from COS cell membrane was further increased by ±57% (Fig 8A, lanes 3), suggesting that LOX-1 receptor is a substrate prone to the activity of different additional proteases.

Fig 8B shows the inhibitor screening for sLOX-1 release induced by the treatment of COS cells with EA cells conditioned medium in the presence of different protease inhibitors. Transfected COS cells were incubated for 10 minutes with fresh DMEM (lane 1) or with conditioned medium derived from EA cells (Fig 8B, lane 2). The presence of GM6001 protease inhibitor in the cell-incubation medium completely abolished cell sheddase activity (lanes 3 and 4). Conversely, neither Pepstatin A (10μM) nor PMSF (2mM) (lanes 5 and 6) brought about any significant inhibition of sLOX-1 shedding. These results demonstrate unequivocally that, also in EA cells, shedding is mediated by metalloproteinases, even though we cannot select, at this stage, which member of the class is actually involved in the shedding activity.

**Fig 9:**
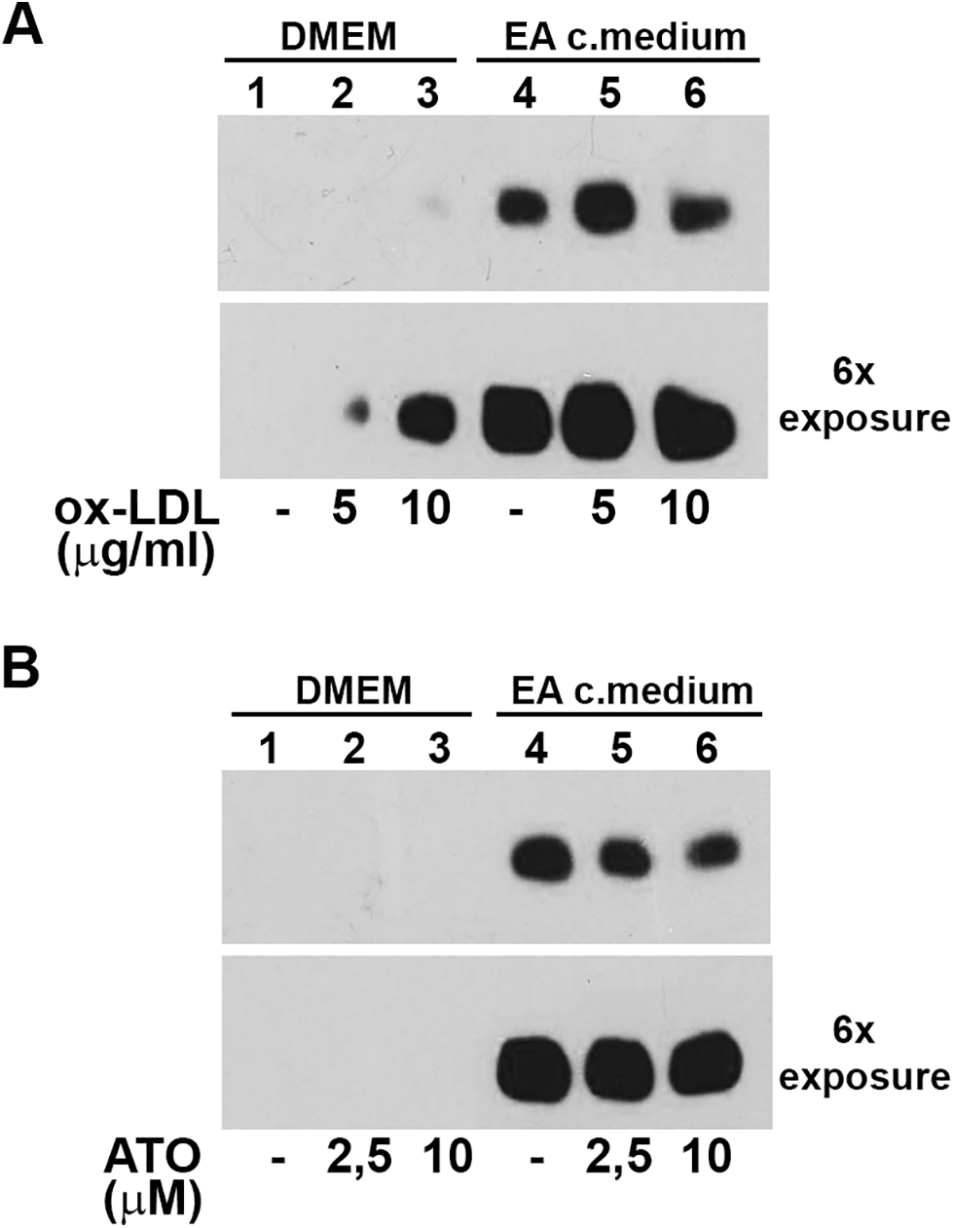
Effect of receptor-ligand on LOX-1 shedding. Western blot analysis of media derived from LOX-1-V5 COS transfected cells incubated for 10 min with serum free DMEM (lane 1–3) or conditioned medium derived from EA cells (lane 4–6), in the presence of ox-LDL (A) and atorvastatin (B) at different concentrations, as indicated. Lower panels in (A) and (B) show a 6 times exposure of the gels.

Since we have shown that ox-LDL-LOX-1 binding induces a cell membrane redistribution of receptors leading to clusterization of LOX-1 receptors in lipid rafts (Matarazzo et al. 2012), we asked whether receptor shedding occurs even when LOX-1 is bound to its functional ligand ox-LDL. Hence, we investigated the influence of ox-LDL ligand on sLOX-1 shedding. Transfected COS cells were incubated for 10 min with fresh DMEM (Fig 9A, lanes 1–3) or EA conditioned medium (Fig 9A, lanes 4–6) in the absence or in the presence of ox-LDL, at concentrations that completely saturate LOX-1 binding sites. As it can be seen, a very low amount of sLOX-1 was released after 10 min incubation with DMEM, and, in the presence of ox-LDL (Fig 9A, lanes 2 and 3), an increase in band intensity becomes detectable whenever the Western blots were exposed 6 times longer (Fig 9A, lanes 2 and 3, lower panel). Conversely, the addition of ox-LDL to EA conditioned medium did not change the amount of sLOX-1 shed (Fig 9A, lanes 5 and 6). The sheddase activity is accomplished also when the receptor is masked by its bulky ligand (*i*.*e*., ox-LDL), suggesting that receptor occupancy might even slightly promote sLOX-1 shedding. Of note, treatment with 2.5 and 10μM atorvastatin for 10 min, which directly binds the hydrophobic tunnel to LOX-1 dimer [23], does not influence sLOX-1 shedding even when blots were exposed 6 times longer (Fig 9B).

### Soluble MMPs mediate LOX-1 receptor shedding *in vitro*

**Fig 10:**
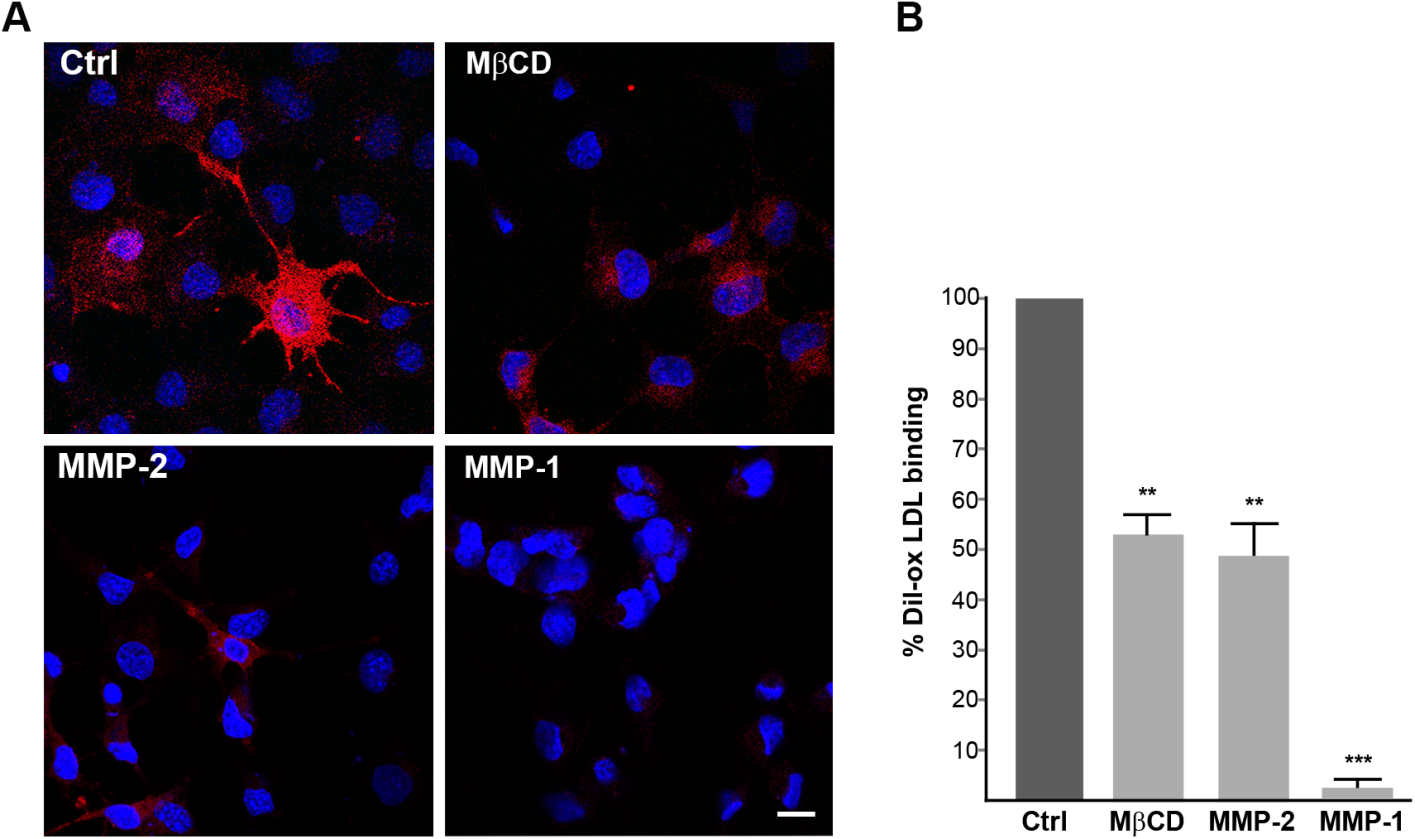
Soluble MMPs mediate LOX-1 shedding. (A) Fluorescence analysis of LOX-1-V5 transiently transfected COS cells treated without (Ctrl) or 5 mM MβCD or 30μM active MMP-2 or active MMP-1 for 1 h at 37°C. Images show Dil-labelled ox-LDL (red fluorescence) for 1 h at 4°C. Nuclei are blue stained with Hoechst 33342. Scale bar 20 nm. (B) Histogram shows the percentage of red positive cells in different treatments, counting Hoescht-stained nuclei (n≥150). Data represent the average ± SEM of two different experiments.

Ox-LDL-mediated enhancement of the MMP proteolytic capability of EA cells secretome suggests that endothelial conditioned medium contains a soluble protease, responsible for LOX-1 shedding. To validate whether a purified soluble MMP was capable of sLOX-1 sheddase activity *in vitro*, we developed a cell-based sheddase assay for LOX-1 receptor. Among the secreted MMP members, we selected collagenase MMP-1 and gelatinase MMP-2, which are up-regulated in abnormal arteries and in heart-failure patients, respectively [42, 48]. The purified MMP was chemically activated and supplied at a certain titrated concentration to transiently transfected COS cells expressing LOX-1-V5 (see MM section for details). Cell fluorescence microscopy technique was employed to visualize LOX-1 receptors “in situ” on individual cells. The key advantage of this approach is that the proteolytic activity validation occurs on a functional substrate receptor exposed on the cell membrane of living cells. A fluorescence labelled Dil-ox-LDL was then used to visualize LOX-1 receptors at the cell surface. After 1 hour incubation of living-labelled cells with 30μM of active-site titrated MMP, cells were incubated with Dil-ox-LDL for 1 hour at 4°C, fixed and analysed. DiI-ox-LDL efficiently binds to LOX-1 receptors in control cells and representative images of Dil-ox-LDL binding are shown in Fig 10A. Of note, MβCD causes a marked loss of specific ox-LDL binding, making the typical membrane fluorescence more diffuse and less intense, as described previously [20]. Similarly, cell exposure to either MMP-1 and/or MMP-2 active enzyme blunted cell fluorescence signal. In the case of the gelatinase MMP-2 50% of the fluorescence signal was dumped, the effect being comparable to that shown by the MβCD lowering drug. At the same stoichiometric concentration of MMP-2 active site, active MMP-1 collagenase shows an even higher LOX-1 degrading capacity, only 5% of cell fluorescence being retained. Therefore, *in vitro*, a soluble active MMP can shed LOX-1 membrane receptor bound to ox-LDL in living cells.

## Discussion

A number of studies have demonstrated that the content of membrane cholesterol can alter the function of receptors, through cholesterol-protein interactions and by changes in membrane viscosity [31,35,36]. Thus, many receptors are preferentially found inside cholesterol-enriched domains, leading to the hypothesis that these domains can organize ligand-induced signalling molecules in the membrane to inhibit specific events [61,62,63]. Previously, we reported that LOX-1 receptors can be down-regulated by either the cholesterol-sequestering agent (*i*.*e*., MβCD) or by cholesterol synthesis inhibitors (*i*.*e*., statins) [20]. In order to clarify how LOX-1 membrane removal occurs, we have employed V5-tagged LOX-1 receptors expressed in HEK-293 or COS cells, and EA endothelial cells as cell model systems to follow up the physiological response to the ox-LDL-LOX-1 binding.

In this study, we demonstrate that membrane cholesterol-lowering drugs induce membrane removal of LOX-1 surface receptors, through two distinct secretory pathways depending on the cell type, namely i) as full length LOX-1 receptor within exosome membranes, or ii) as a truncated soluble fragment sLOX-1, which results from proteolytic shedding of the receptor ectodomain. The relevance of the whole LOX-1 removal seems minor for the atherosclerotic plaque evolution as a very low amount of full-length LOX-1 was found associated to exosomes in the secretome of human EA endothelial cells. On the other hand, LOX-1 receptor shedding process, producing sLOX-1, seems relevant for the elucidation of the role played by LOX-1 in endothelial cell membranes for the development of atherosclerosis. As a matter of fact, we find that membrane cholesterol modulates LOX-1 shedding in all types of cells expressing LOX-1 (*i*.*e*., human EA-hy926 cells, HEK-293#19, CHO-F2 cells and transiently transfected COS cells), even though to a different extent. Its relevance is confirmed by the evidence that significant levels of the soluble form of LOX-1 can be measured *in vivo* in serum of patients affected by cardiovascular diseases. Thus, the amount of sLOX-1 increases in atherosclerosis and inflammation and it has been identified as a biomarker for early diagnosis of acute coronary syndrome [14,15].

An important aspect, which appears correlated with sLOX-1 shedding, is the structural arrangement of the cell membrane. Exposure of the cells to MβCD, which reduces membrane cholesterol content and disrupts lipid rafts and caveolae, indeed facilitates sLOX-1 shedding. This outcome seems to be related to our previous observation that cholesterol promotes LOX-1 clustering in lipid raft [20], envisaging the possibility that clustering of LOX-1 in lipid rafts protects LOX-1 from proteolytic attack. This behavior must be then correlated with the regulation of the shedding process, since, for the cell lines investigated, we have shown that LOX-1 shedding, triggered by cholesterol depletion agents, is a metalloproteinase-dependent process, even though the identification of the specific metalloproteinase(s) is not straightforward. Thus, among MMP members, MMP-1, MMP-2 and MMP-3 appear to be more consistently correlated with the cardiovascular risk (Newby, 2015), while MMP-1, MMP-3 and MMP-9 turn out to be up-regulated in atheromatous endothelial cells [42,64]. Previous works report that transfected cells with ADAM members undergo a cholesterol-dependent redistribution of several membrane proteins associated to the proteolytic shedding of APP [29], interleukin-6 receptor (Matthews et al. 2003) and CD30 [36]. Further, membrane-anchored MMPs, such as MT1-MMP (*e*.*g*., MMP-14), have been reported to be able to interact with LOX-1 [65,66,67,68]. An additional element, playing an important role, is the balancing between MMPs and TIMPs levels. Indeed, an unbalanced ratio of MMPs/TIMPs serum levels has been reported to be associated to varieties of cardiovascular diseases [47] and statins treatments of subclinical or clinical heart-failure patients have been shown to revert the MMPs/TIMPs serum levels [48]. Consistently, we show in this paper that EA cells show a remarkable enhancement of soluble MMPs levels (namely MMP-1, MMP-2 and particularly MMP-9) upon ox-LDL binding to LOX-1, which is also associated to a decrease of TIMP-1 secretion level (Fig 6) and to the consequent increase of MMP activity.

An additional, very important and novel outcome here reported is the finding that, *in vitro*, endothelial cells secrete (a) soluble mediator(s), which induces LOX-1 ectodomain shedding in crossed-cells experiments. Therefore, the results presented here show for the first time that a soluble factor, present in endothelial secretome, can induce a naive cell to shed LOX-1 (Fig 7). Consistently, the results of cell-fluorescence assay confirm that, also *in vitro*, sheddase activity can be played by a soluble enzyme (such as MMP-1 and MMP-2), indicating that receptor shedding may be modulated not only by membrane-anchored proteases, but also by (a) soluble factor(s) which may be released even by an additional cell. This is particularly relevant since, up to now, only two membrane proteases were reported to be possible candidates for LOX-1 sheddase, namely i) ADAM10, as IL18 stimulated HEK-293 cells transfected with ADAM10 increase LOX-1 shedding [69], and ii) MT-MMP1 as fluorescent immunostaining of HAEC cells revealed its partial co-localization with LOX-1 [66]. Even though we cannot rule out the possibility that additional members of the MMP and/or ADAM family are involved in LOX-1 sheddase activity, we can speculate that the LOX-1 sheddase-system is more redundant than previously thought, envisaging the possibility that a) different proteases can mediate the same shedding process, and b) a given cell can possibly employ different sheddase(s) depending on the specific biological stimuli.

In conclusion, our findings indicate that the shedding secretory pathways of LOX-1, which is markedly displayed in endothelial cells, is metalloproteinase-dependent and that it can be performed by a soluble MMP, such as MMP-1 collagenase or MMP-2 gelatinase, even though also additional and important member(s) of the metalloproteinase family might be able to shed LOX1 receptor. Further, we have demonstrated that the sLOX-1 shedding occurs on the ligand-free receptor and the presence of ox-LDL only slightly interferes with sLOX-1 shedding; thus, neither the physiological ligand ox-LDL nor a drug ligand, such as atorvastatin [23] (Fig 4A, lane 5, and Fig 8B) prevent the sLOX-1 shedding. Overall, the sheddase(s) cleave(s) the free or the ligand-bound ectodomain at the stalk/linker region, thus regulating the pool of active receptor available at the cell surface for ligand-induced signalling. Further studies are required to identify and characterize the full spectrum of metalloproteinases that cleave LOX-1 in various cellular systems under constitutive and/or ox-LDL stimulated conditions.
